# Large-Scale Information Flow in Conscious and Unconscious States: an ECoG Study in Monkeys

**DOI:** 10.1371/journal.pone.0080845

**Published:** 2013-11-15

**Authors:** Toru Yanagawa, Zenas C. Chao, Naomi Hasegawa, Naotaka Fujii

**Affiliations:** Laboratory for Adaptive Intelligence, Brain Science Institute, RIKEN, Saitama, Japan; Cuban Neuroscience Center, Cuba

## Abstract

Consciousness is an emergent property of the complex brain network. In order to understand how consciousness is constructed, neural interactions within this network must be elucidated. Previous studies have shown that specific neural interactions between the thalamus and frontoparietal cortices; frontal and parietal cortices; and parietal and temporal cortices are correlated with levels of consciousness. However, due to technical limitations, the network underlying consciousness has not been investigated in terms of large-scale interactions with high temporal and spectral resolution. In this study, we recorded neural activity with dense electrocorticogram (ECoG) arrays and used the spectral Granger causality to generate a more comprehensive network that relates to consciousness in monkeys. We found that neural interactions were significantly different between conscious and unconscious states in all combinations of cortical region pairs. Furthermore, the difference in neural interactions between conscious and unconscious states could be represented in 4 frequency-specific large-scale networks with unique interaction patterns: 2 networks were related to consciousness and showed peaks in alpha and beta bands, while the other 2 networks were related to unconsciousness and showed peaks in theta and gamma bands. Moreover, networks in the unconscious state were shared amongst 3 different unconscious conditions, which were induced either by ketamine and medetomidine, propofol, or sleep. Our results provide a novel picture that the difference between conscious and unconscious states is characterized by a switch in frequency-specific modes of large-scale communications across the entire cortex, rather than the cessation of interactions between specific cortical regions.

## Introduction

As humans, we experience stable consciousness while we are awake but easily lose this conscious state during sleep. One intriguing hypothesis aimed to explain the neural mechanism underlying consciousness and unconsciousness, is that neural integration within the brain generates the conscious state, while the loss of the integration is responsible for producing the unconscious state [1-4]. This “integration theory” has been evaluated by quantifying neural interactions in the brain, and previous studies have confirmed the reduction of neural interaction occurring under the unconscious state by using various measurements ranging from functional imaging [5-7] to electroencephalography (EEG) [8-11]. In the spectral domain, the reduction of neural interactions in the unconscious state occurs specifically in the gamma oscillation range [8]. Previous studies have also demonstrated the importance of specific region interactions during the conscious state, which include thalamus-frontoparietal [5,6], frontal-parietal [7,9,11,12], and parietal-temporal interactions [7]. Additionally, compared to bottom-up interactions, top-down interactions from the frontal to the parietal cortices [9,11,12] were more correlated with the conscious state. 

However, due to several technical limitations, such as low temporal-resolution in blood oxygen level-dependent signal and low spatial resolution with narrow spectral range in EEG, previous studies have failed to explore these neural interactions at higher temporal and spatial resolution and over wider frequency bands.

In order to overcome the existing technical limitations and map the global neural interactions correlated with conscious and unconscious states, we developed a novel recording method—the multi-dimensional recording (MDR) technique [13]. In MDR, neural activity can be recorded via ECoG at high temporal (>1 KHz) and spatial (~3 mm) resolution, allowing for the measurement of precise phase synchrony in terms of global network causality with high temporal stability [14]. To our knowledge, ECoG is the most balanced technology for obtaining detailed recordings of global neural interactions. 

In this study, we extracted the comprehensive consciousness related network based on ECoG signals that were recorded from most of the lateral cortex in 4 macaques during awake, anesthetized and sleeping states. We identified large-scale network components in spectral domains, which corresponded to conscious and unconscious states. In contrast with previous results, our result that the modulation of cortical interactions correlated with conscious states was not limited within specific cortical regions, but was found in all combinations of cortical region pairs. We found that neural interactions between cortical regions that fell into a specific spectral domain drastically changed between conscious and unconscious states, indicating that the unique frequency-specific modes of large-scale region interactions over the entire cortex might be the fingerprint of conscious versus unconscious states. 

## Results

### Cortical interactions

**Figure 1:**
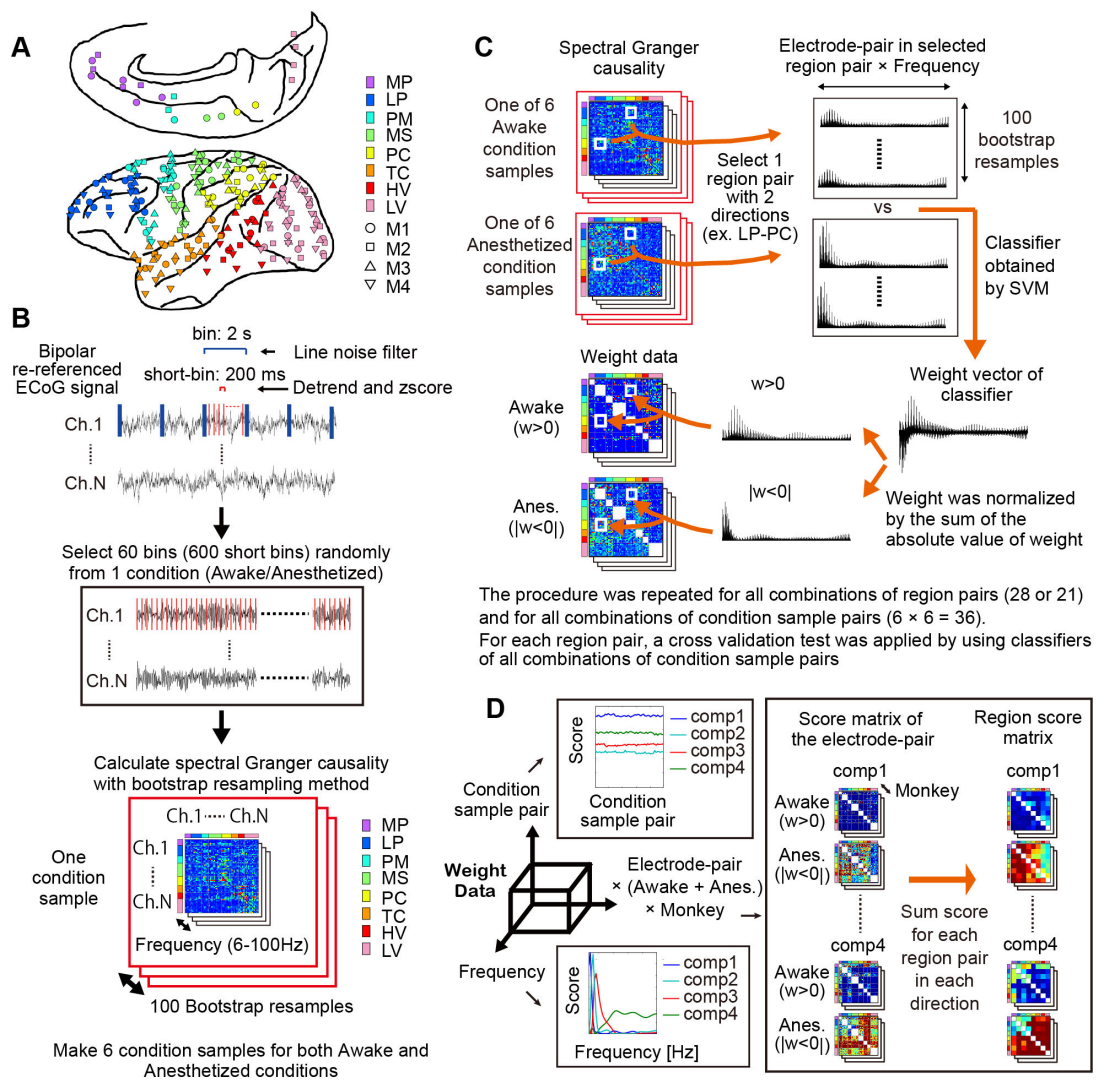
Schematic explanation of method. A: The bipolar re-referenced ECoG electrode arrays on the left cortical surface of 4 monkeys (M1-M4). Marker colors represent cortical regions, and shapes of markers represent monkeys. MP: Medial prefrontal cortex LP: Lateral prefrontal cortex, PM: Premotor cortex, MS: Primary motor and somatosensory cortices, PC: Parietal cortex, TC: Temporal cortex, HV: Higher visual cortex and LC: Lower visual cortex. The positions of the ECoG electrode in monkeys M1, M3, and M4 were manually remapped on the brain of monkey M2 by using the sulci as remapping references. B: Preprocessing of ECoG signals for spectral Granger causality and its data structure. For preprocessing of the ECoG signal, bipolar re-reference, line noise filter, detrend, and z-score transformation were applied. Next, 120-sec time length data was prepared as a condition sample from the data set collected on different experimental days. One hundred bootstrap resamples of spectral Granger causalities were calculated from 1 condition sample. Six condition samples were prepared for the awake and anesthetized conditions. C: The procedure for obtaining the weight data for the awake and anesthetized conditions. SVM was generated to classify bootstrap resamples of spectral Granger causality between the awake and anesthetized conditions for all combinations of region pairs and for all combinations of condition sample pairs. Weights of the classifier for all combinations of region pairs were separated for the awake and anesthetized conditions based on their sign and were integrated in the weight data. D: By using PARAFAC, network components were extracted from the global pattern of the weight data. The weight data of 4 monkeys were combined and aligned to 3 dimensions, 1) condition sample pairs, 2) frequency, and 3) electrode pairs of all combinations of region pairs for awake and anesthetized. After the deconstruction, the components in each dimension had relative scores.

We first focused on the difference in cortical interaction between awake and ketamine–medetomidine-induced anesthetized conditions. To extract/construct the sustained network of neural interactions from the recorded neural data, we pooled the ECoG data with a 120-sec time length (200 ms × 600) from the data set collected on different experimental days, and calculated a single model of spectral Granger causality for all of individual pairs of bipolar signals (See Figure 1B and methods). Granger causality [15,16] was calculated with a model order of 7 (35 ms) for each pair (total 62 × 61 = 3,782 pairs for monkeys M1 and M2, and 63 × 62 = 3,906 pairs for monkeys M3 and M4) of bipolar channels during the awake and anesthetized conditions, at 48 frequency windows (6 to 100 Hz, separated every 2 Hz without overlap). We regarded a data set with a 120-sec time length as 1 condition sample, and prepared 6 condition samples for the awake and anesthetized states. By using the bootstrap resampling methodology, 100 samples of spectral Granger causality were calculated for each condition sample (See Figure 1B). 

To extract significant inter-region interactions, bipolar electrodes were manually grouped into 8 cortical regions (Figure 1A). We used a support vector machine (SVM) [17] for each combination of cortical region pairs (28 pairs for monkeys M1 and M2, and 21 pairs for monkeys M3 and M4) and for each combination of condition samples (6 × 6 = 36 pairs) in order to categorize the bootstrap resamples of spectral Granger causality into awake and anesthetized conditions (Figure 1C). 

For a cross validation, the classifier which was trained by each condition sample pair was tested by the other condition samples which were not used to train the classifier. The accuracy of each classifier was calculated from the rate of correct prediction in the test. To test the statistical significance of each inter-region interaction, we also constructed classifiers from a shuffled condition of the labels for the awake and anesthetized states and the Wilcoxon rank sum test was applied to the classifier accuracies of the 36 condition sample pairs in normal and shuffled conditions (p < 0.05, FDR correction). Final accuracy of each cortical region pair was calculated by averaging classifier accuracies of all condition sample pairs. 

**Figure 2:**
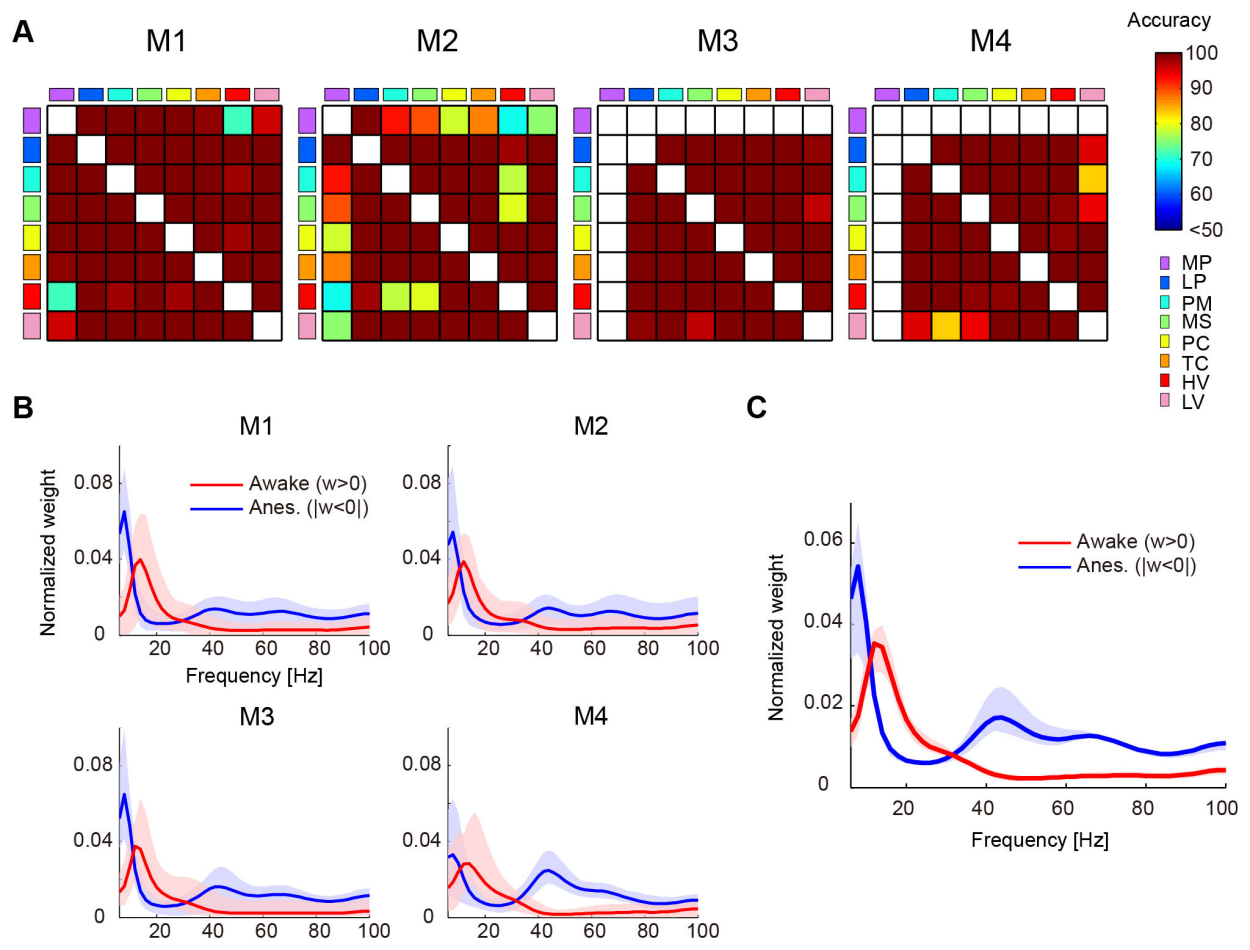
Accuracy of classifier between awake and anesthetized conditions and spectral distribution of classifier weight. A: Accuracy of the classifier for all combinations of cortical region pairs for the 4 monkeys. White squares mean lack of data where no classifier was built. When the accuracy of a region pair was not significant, the accuracy was set to 50%. B: The distribution of normalized weights for the awake (red) and anesthetized (blue) conditions averaged for all combinations of cortical region pairs. For preprocessing, the weight data of each region pair was normalized by the sum of the absolute value of weight and was averaged for all combinations of condition sample pairs. The normalized weights were averaged for all combinations of cortical region pairs and are shown in the line. The range from minimum to maximum values in cortical region pairs is shown by the shading around the line. C: The distribution of averaged weights of region pairs for the awake (red) and anesthetized (blue) conditions for the 4 monkeys. The averaged distribution of the 4 monkeys is shown by a line plot, and the range from minimum to maximum values is indicated by the shading around the line.

For all combinations of cortical region pairs, the classifier predicted the awake and anesthetized conditions from the spectral Granger causality with significant accuracy (Figure 2A). This indicates that the modulation of neural interaction occurred over the entire cortical surface by manipulating conscious states. 

### Interactive Band Shift in the Spectral Domain

We examined the weight distribution of the classifiers in the spectral domain. Since we were interested in relative weight patterns in the spectral domain, the weight of the classifier of each region pair was divided by the sum of the absolute value of the weight in the classifier for normalization. Since spectral Granger causality is always positive, positive weight was associated with the awake condition (w > 0), while negative weight was associated with the anesthetized condition (|w < 0|). We then separated weights based on their sign (either positive or negative) and, in the following analysis, used them as the weight for the awake and anesthetized conditions, respectively (Figure 1C). Distribution of weights in the spectral domain shared a similar pattern amongst all combinations of region pairs (Figure 2B), and distribution of the averaged weights for all region pairs was consistent amongst the 4 monkeys (Figure 2C). In the distribution, alpha and beta bands (12–32 Hz) had higher weights for the awake condition. On the other hand, the theta (6–10 Hz) and gamma (34–100 Hz) bands had higher weights for the anesthetized condition. This suggests that the band of informative interaction in the spectral domain shifts dynamically between altering states of wakefulness.

### Network components of awake and anesthetized states

Since the accuracy of the classifier was significant for all combinations of cortical region pairs (Figure 2A), we focused on the global pattern of the weights. 

The normalized weights of each classifier responsible for discriminating between awake and anesthetized conditions were separately integrated into the weight data (Figure 1C). The total pattern of the classifier weights formed a network since each of the weights was assigned to all pairs of electrodes. We then extracted latent structures of the network from the weight data by using Parallel factor analysis (PARAFAC, Figure 1D). PARAFAC is a method that fits multiple arrays simultaneously in terms of a common set of factors differing in relative weights in each dimension and have been applied to EEG study [18]. The weight data was aligned to 3 dimensions: condition sample pair for the first dimension, frequency for the second dimension, and all combinations of region electrode pairs for the third dimension (Figure 1D). In the third dimension, the electrode pairs for the awake and anesthetized conditions were combined for all 4 monkeys.

**Figure 3:**
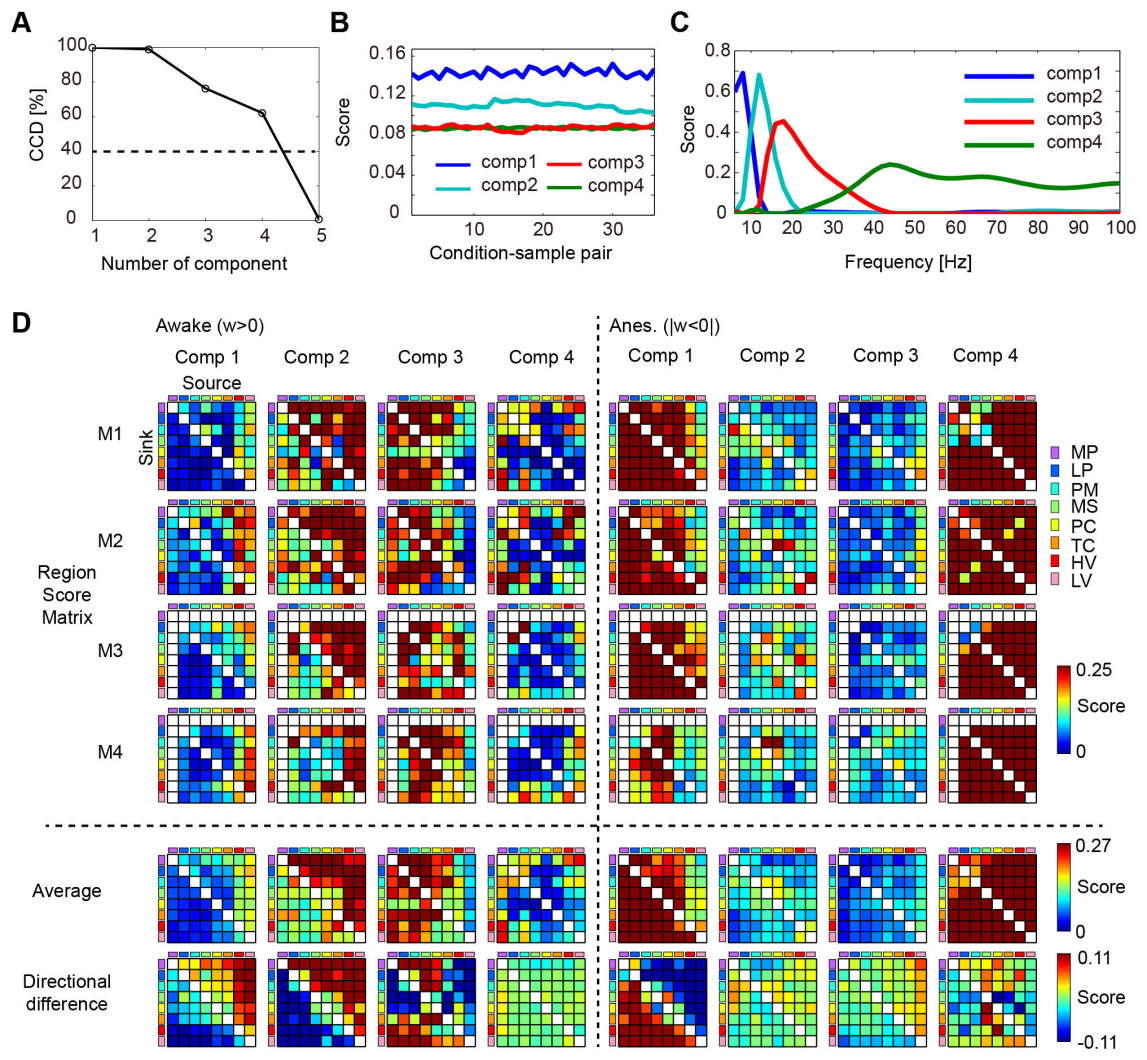
Network components extracted from the global pattern of the weight data by using PARAFAC. A: CCD for the number of components. The threshold was set to 40%. B: The scores for the dimension of condition sample pairs. C: The scores for the dimension of frequency. D: (M1-M4): The region score matrix for the awake and anesthetized conditions and for the 4 monkeys. (Average): The region score matrix was averaged for the 4 monkeys. (Directional difference): The difference of the scores between the 2 directions was calculated for each region pair.

The reliability of the PARAFAC model was evaluated by Core consistency diagnostics (CCD), which automatically suggested the proper number of components [19,20]. We found that the weight data could be divided into 4 components (Figure 3A). Constraints on dimension in PARAFAC were set to non-negativity for 3 dimensions; thus all values of score were always positive. Since all values in the weight data were positive, scores had a positive correlation with weights. Figure 3B shows the score of condition sample pairings. The scores were nearly constant irrespective of condition sample pairs for each component, which indicates that the difference between the awake and anesthetized conditions was consistent amongst varying combinations of condition sample pairs. Figure 3C shows the score distribution in the frequency domain. Each component showed a unique peak in the frequency that was 8, 12, 18, and 44 Hz. For convenience, we will refer to the bands of the 4 components as theta, alpha, beta, and gamma.

Figure 3D shows region pairing scores for the 4 monkeys, in which scores of electrode pairs were summed for each region pair in terms of interaction direction (Figure 1D). Since region matrix scores demonstrated consistency across the 4 monkeys (Figure 3D, M1–M4 and see Figure S5 for the quantification of the individual consistency), the scores were averaged for each component (Figure 3D, Average), and the difference between the 2 interaction directions of cortical region pairs in the averaged score matrix was calculated (Figure 3D, Directional difference). The second and third components were comprised of large weights for the awake condition while the first and fourth components were comprised of large weights for the anesthetized condition (Figure 3D, Average).

The interactions of the first to third components showed direction biases (Figure 3D, Directional difference), while the fourth component exhibited very minor bias in direction. The interaction in the top-down direction (anterior to posterior) was dominant in the first component, while the interaction in the bottom-up direction (posterior to anterior) was dominant in the second component. In the third component, the interactions amongst the top-down and bottom-up directions were mixed. Lastly, the fourth component had a lack of bias in either top-down or bottom-up directions (Figure 3D, Directional difference).

**Figure 4:**
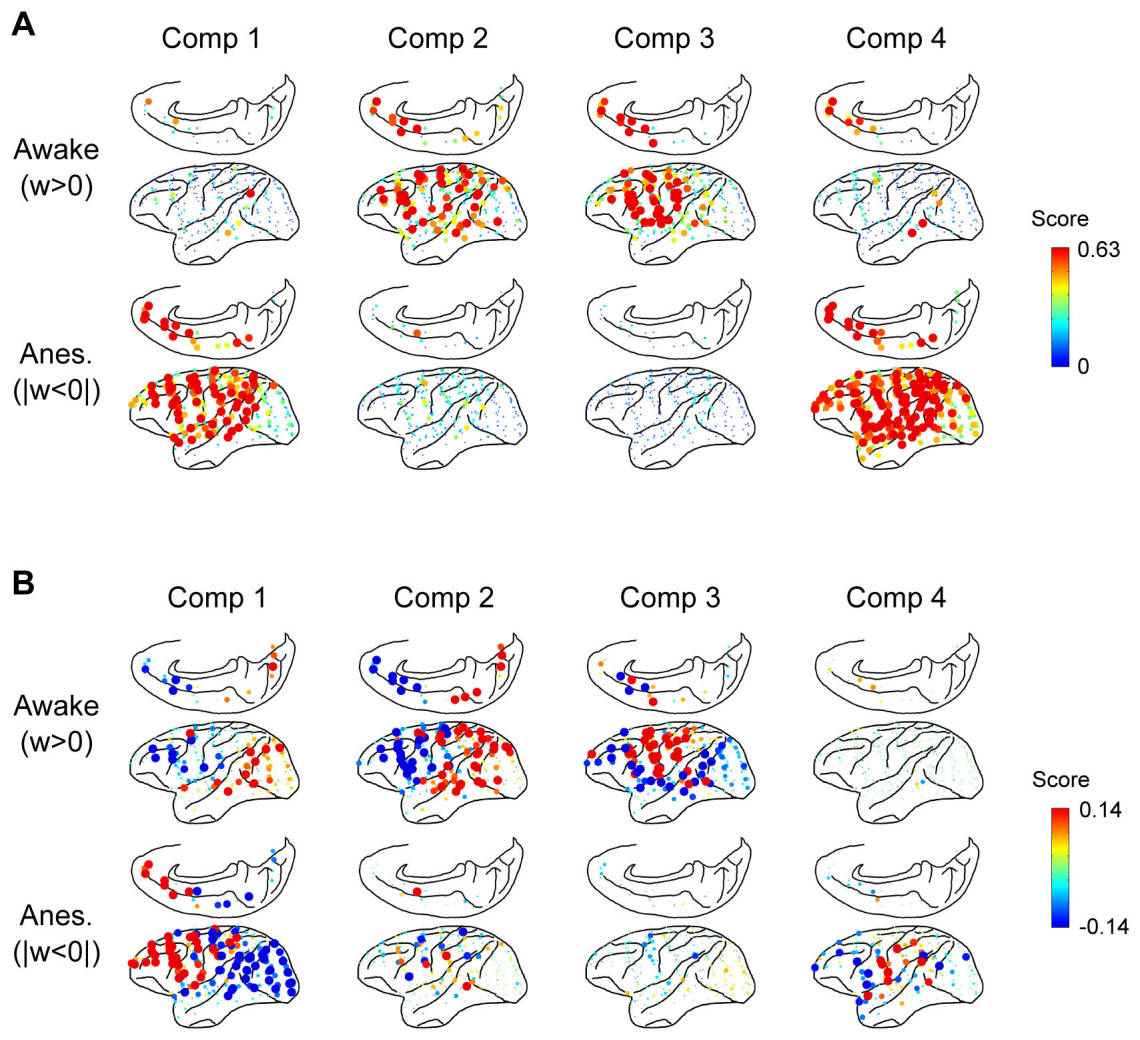
Spatial distribution of electrodes for information outflow and inflow in 4 network components. A: The distribution of electrode scores was summed for 2 interaction directions. The value was calculated for each component and for awake and anesthetized conditions. A high score indicated that the electrode had very high interaction with other electrodes. The values for the 4 monkeys were superimposed on 1 brain map. B: The distribution of electrode scores was summed for each interaction direction, and the difference between the 2 interaction directions was calculated for each component and for awake and anesthetized conditions. The red dot indicates that the information outflow of the electrode was larger than information inflow, and the blue dot indicates vice versa. The values for the 4 monkeys were superimposed on 1 brain map.

In order to visualize the fine structure of the components at the electrode level, the matrix score of electrode pairs in 2 interaction directions was summed (Figure 4A), and the difference between the interaction directions was calculated for each electrode (Figure 4B). In the first component for the anesthetized condition, the outflow of information was dominant in the frontal regions (red dots in Figure 4B, Comp1, Anes.). On the other hand, the inflow of information was dominant in the temporal and visual cortices (blue dots in Figure 4B, Comp1, Anes.), and this trend was reversed in the second component in the awake condition (Figure 4B, Comp2, Awake). In the third component of the awake condition (Figure 4B, Comp3, Awake), the dominant region of information outflow (red dots) was found in the somatosensory motor cortices that sunk in surrounding areas (blue dots). Lastly, in the fourth component of anesthetic condition, there was no apparent cluster (Figure 4B, Comp4, Anes.).

### Universality for different unconscious states (anesthesia and sleep)

**Figure 5:**
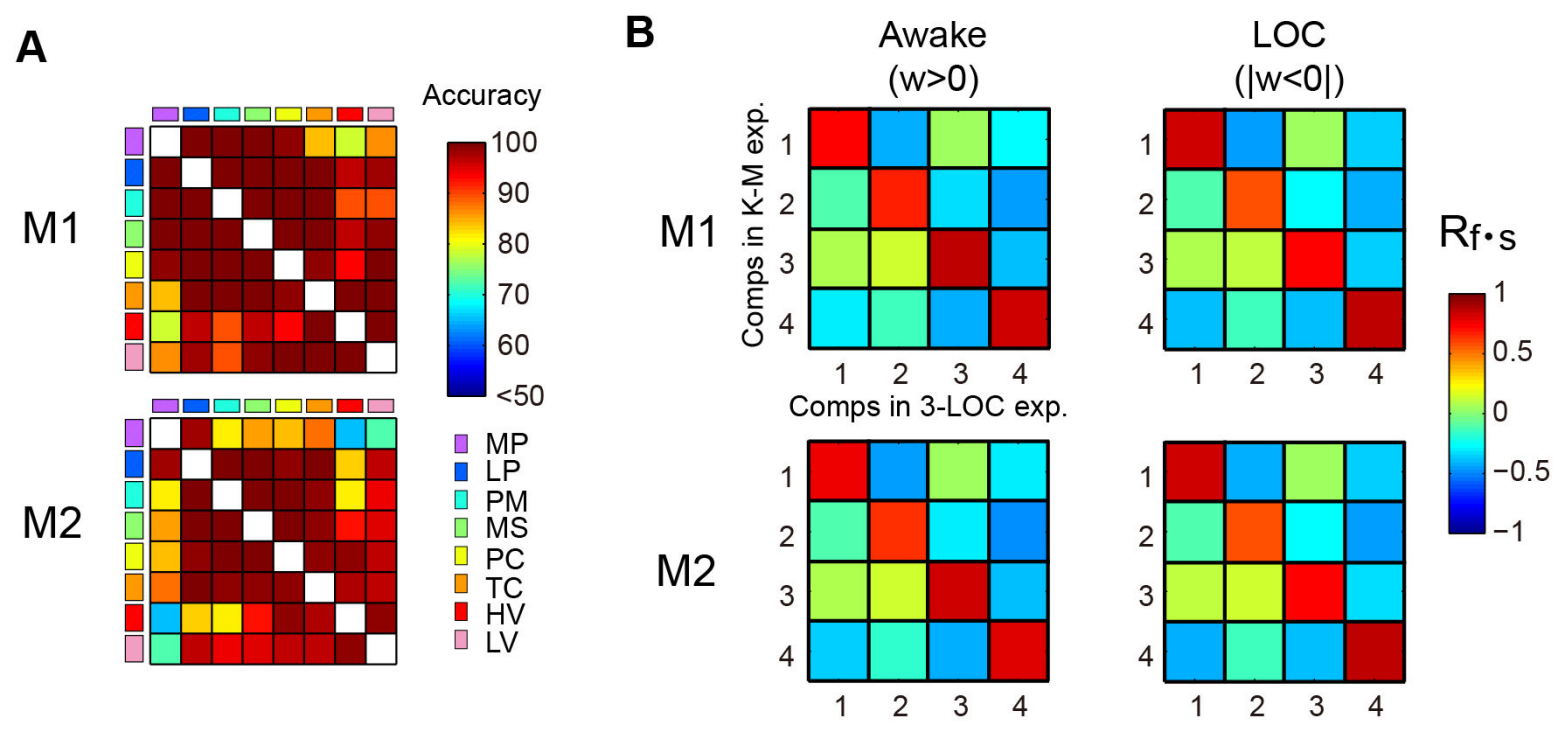
Similar accuracy of classifier and network component between ketamine–medetomidine and 3-LOC experiments. A: Accuracy of the classifier to categorize the awake and LOC conditions for all combinations of cortical region pairs for monkeys M1 and M2. Figure format is the same as Figure 2A. B: Spatial-frequency correlations between components in the ketamine–medetomidine (K-M) experiment and that in the 3-LOC experiments. The correlation was calculated for monkeys M1 and M2 and for the awake and LOC conditions. The spatial-frequency correlation evaluated the similarity in pattern of components in the dimensions of frequency and electrode pairs.

In order to verify the universality in modulation of interacting network patterns responsible for characterizing awake and anesthetized conditions, we tested network interactions in different unconscious states. In order to do this, we conducted 2 additional experiments: 1) propofol-induced anesthesia and 2) natural sleep. The propofol-induced anesthesia experiment consisted of awake and anesthetized conditions, whereas the sleep experiment consisted of awake and natural sleep conditions. Spectral Granger causality was calculated for each of the additional experiments. For each condition, the bootstrap samples of Granger causality of ketamine–medetomidine- and propofol-induced anesthesia as well as natural sleep (3-LOC) experiments were combined to form the “loss of consciousness (LOC)” condition, and spectral Granger causality of the awake condition of 3-LOC experiments were combined under the “awake” condition. The classifier to predict awake and LOC conditions was then constructed. We combined spectral Granger causality of the different experiments because we were interested in the common neural interactions contributing to the discrimination of the awake and LOC conditions. Figure 5A shows that the classifier accuracy was significant for all combinations of cortical region pairs (p < 0.05, FDR correction). This indicates that we successfully constructed a universal classifier capable of separating awake and LOC conditions. 

The weight data of the classifier in 2 monkeys was combined and deconstructed into its components by PARAFAC. We found a similar pattern of 4 components in the dimensions of condition sample pair, frequency, and electrode-pairs of all combination of region pairs for the awake (w > 0) and LOC (|w < 0|) conditions. To prove the similarity, we defined a spatial-frequency correlation as:

in which R_f_ is the spearman correlation coefficient of scores of components in the frequency dimension between the ketamine–medetomidine and 3-LOC experiments. R_s_ is the spearman correlation coefficient of scores of components in the electrode-pair dimension between the ketamine–medetomidine and 3-LOC experiments. R_s_ was calculated for each monkey and for the awake (w > 0) and LOC (|w < 0|) conditions. Figure 5B shows that each component in the 3-LOC experiments had high spatial frequency correlation with 1 component in the ketamine–medetomidine experiment for each monkey, which indicates that the classifier in the 3-LOC experiments is similar to that in the ketamine–medetomidine experiment with regard to in conscious and unconscious patterns. These results indicate that the difference of the neural interactions between the awake and unconscious conditions could be generalized in the anesthetized and sleep states, and could be deconstructed into 4 network components.

## Discussion

### Whole cortical interactions

In previous studies, large scale analyses targeted consciousness-related interactions amongst brain areas confined to specific inter-regions such as the thalamus-frontoparietal [5,6], frontal-parietal [7,9,11,12], and parietal-temporal [7] regions. In this study, we found that significant modulation of neural interaction discriminates between conscious and unconscious states in all combinations of cortical region pairs, and not only in specific region pairs (Figure 2A and 5A). The advantage of our recoding method is that it allowed us to record signals with high temporal and spectral resolution from most cortical regions. In analytical method, we used spectral Granger causality with a wide range of frequency bands in the pattern classification analysis. Unlike other recording and analytical methods, those techniques allowed us to extract more information so that we could classify conscious and unconscious states with better performance. Specifically we were able to identify unique frequency bands, which were informative for discriminating between conscious and unconscious states (Figure 2B and 2C).

### Technical Advantages and Limitations of Granger Causality

Although spectral Granger causality is the useful method for measuring neural interactions, there are some technical limitations. One is that spectral Granger causality measures bivariate causality so that it could extract the false interaction between two nodes when there was no actual interaction but they were influenced by a common source. In the current analysis, we cannot exclude the possibility. The multivariate autoregression model, which is the extension of Granger causality concept, is one option to overcome the problem by taking account of the multivariate dependencies of signals [21-23]. 

Another limitation is that spectral Granger causality cannot discriminate the functional and effective connectivity [24,25]. The method for measuring the effective connectivity, such as dynamical causal modeling (DCM)[26], have a potential to identify the most likely brain network which explains the observed neural signal. But the number of network node of the model in DCM tends to be sparse [12,27] and so far it is hard to presuppose the plausible network model of large-scale network. In contrast, spectral Granger causality is "model free" technique. In this study, we collected neural data from entire lateral cortex where number of nodes was unknown. Thus, even though there was the possibility of picking false interaction, “model free” spectral Granger causality has certain advantage as a first step to reveal comprehensive consciousness network from the massive dataset [24]. 

### Existence of the unconscious network

In this study, we used the integration theory to hypothesize that the conscious state is generated by highly integrated neural interactions that disappear in the unconscious state [1-4]. Our results half supported this hypothesis. We identified 4 network components that contributed to conscious and unconscious states. The second and third components reduced interactions in the unconscious state in both alpha and beta bands, which supports the integration theory; however, in the first and fourth components, integrated neural interactions in theta and gamma bands emerged under the unconscious state (Figure 2B), which was not predicted by the theory. We defined the second and third components as the conscious network, and the first and fourth networks as the unconscious network. 

A few reports have acknowledged the existence of the unconscious network under anesthesia [28] and epilepsy [29]. Specifically, under anesthesia, Granger causality between the anterior and posterior cingulate cortices has been reported to increase in both beta and gamma bands in human EEG studies [28]. However, the large-scale network interactions that increase under unconscious states have not been systematically examined. Consequently, our findings regarding the unconscious network might be the first report. 

### Unconscious network in the theta band

Slow wave oscillation (0.1–4 Hz) is one of the characteristic features seen in sleep and under anesthesia [30]. This slow oscillation in the sleeping state can be observed anywhere in the cortex [31] and propagates along the anterior to posterior axis in a top-down manner [31,32]. The first component in our current study comprises of interactions in a top-down manner in the theta band. Although the peak frequency of the first component was found at 8 Hz (Figure 3C), the similar direction of information flow and the slow wave oscillation observed in the previous studies indicate that this component was able to capture a part of the network forming slow wave oscillations.

When slow wave oscillation was evoked during propofol-induced anesthesia, neural spiking occurred within a limited window that caused fragmented neural interactions [33]. Thus, the emergence of the unconscious network in the theta frequency band might reflect active disassembly of the conscious network via a perturbation in spike timing activity.

### Unconscious network in the gamma band

The fourth component’s peak frequency fell into the gamma band and was informative in discriminating the unconscious state. Gamma oscillations are associated with a multitude of cognitive functions [34]. It might seem surprising that the fourth component did not have large weights in the awake condition. One possible interpretation of this is that gamma oscillation under the awake condition is highly transient since it reflects a dynamically changing external environment. Our methods extracted stationary interactions, but were not sensitive enough to extract transient ones. The total distribution of region scores for most of the cortical region pairs in the fourth component (Figure 3D, Anes., Comp4) might indicate that the brain has global uniformity in its synchronized activity under the gamma frequency band in the unconscious state. However, in the conscious state, each cortical region generated transient interactions depending on cognitive demands thus losing the global gamma synchrony. This seems to be consistent with the information integration hypothesis, which suggests that the brain might have the capacity to generate a repertoire of different conditions in the conscious state, but that this ability is lost with the loss of consciousness [35]. According to the hypothesis, uniform synchronizations in gamma bands during unconsciousness might be the condition in which the brain loses its capacity to generate a range of different states.

In the anesthetized condition, spike activity was entrained by the phase of the slow wave oscillation [33]. Therefore, the global gamma synchrony observed in the fourth component might reflect the synchronized spike activity entrained by global slow wave oscillations.

### Conscious network

The second and third components had large weights in the conscious states. The score of the frequency domain in the second (at 14 Hz) and third (at 18 Hz) components correspond to alpha and beta bands (Figure 3C). In terms of the direction of the interaction, the second and third components shared a similar feature in which a sink in interaction was observed in the prefrontal cortex (Figure 4B, Awake, Comp.2 and 3); however, the dominant source regions remained different. The second component had its dominant source in the visual and temporal cortices, whereas the third component’s dominant source was located in the somatosensory motor cortices. Additionally, when compared to the second component, the third component comprised of more top-down interactions stemming from the prefrontal to the visual cortex (Figure 3D, Directional difference, Comp 2 and 3).

### Large-scale interactions in the alpha and beta bands

Previous human magnetoencephalography studies have reported a large-scale correlation in alpha and beta frequency bands during the resting state [36,37]. Those previous findings are comparable to our observation of conscious network in terms of the network’s size and frequency profile. However, in contrast to previous studies, our study successfully extracted directional information of the interactions. Additionally, previous studies failed to test if large-scale correlations would be preserved in the unconscious state. In our study, we found that the large-scale interactions in alpha and beta bands were specifically correlated with the conscious state, and not with the unconscious state.

### Consciousness has a bottom-up type interaction

The conscious network has a unique mode of large-scale cortical interaction. One of the characteristic features of this is that the prefrontal cortex serves as a sink region.　Previous studies have mostly focused on top-down interactions between the frontal and parietal cortices that characterize the conscious state. Compared with the resting conscious state, anesthetized states show the reduction of feedback connectivity in the frontal to parietal network, as reported by human EEG studies [9,11,12]. However, the fact that large disruptions in the frontal cortex do not affect the conscious state [38] indicates that, at least, the information outflow from this region may not be crucial to maintain consciousness. In contrast with the previous studies, our study shows that in the second and third components, was mainly an inflow region (Figure 4B, Awake, Comp 2 and 3), and cortical interactions from sensory and motor cortices converged on the lateral and medial prefrontal cortices. One possible explanation for this reversal in direction might be the difference of signal quality in the spectral domain between EEG and ECoG. Compared to EEG signal, ECoG signal has better spatial resolution and provides more information in the spectral domain, and since the previous studies did not evaluate connectivity in the spectral domain, we cannot directly compare the results. 

### Top-down interactions in the conscious network

Even though bottom-up interactions were dominant in the conscious network, we also found top-down interactions in the third component. Here, we saw that the prefrontal cortex showed top-down interactions with the temporal and lower and higher visual cortices (Figure 3D, Directional difference, Comp 2 and 3). Some studies have reported the presence of top-down interactions in a slow frequency band during an attention task. These studies found the top-down interactions at 4–12 Hz from the visual association cortex to primary visual cortex [39] and at 6–16 Hz from the prefrontal to posterior parietal cortices [40]. In our study, the third component peaked at 18 Hz (Figure 3C), which was a little higher than the values reported by the previous studies. Additionally, in contrast to the previous studies that demonstrated these interactions during an attention task, our study demonstrates the interactions in the resting-state; therefore, the top-down interactions from the prefrontal to visual cortices in the third component cannot be attributed to processing of external visual information. This suggests that the top-down interaction we observed in the third component might reflect baseline information flow occurring in the conscious state, regardless of visual processing.

### Functional significance of the conscious network

Alpha and beta band oscillations in the resting state have been proposed to reflect an idle state [41,42]. However, recent evidence has suggested that alpha and beta oscillations might serve to actively suppress cortical activity in order to regulate information transmission [43,44]. In the theory, the region that was irrelevant to the task being performed was shut down by sustaining a higher level of alpha and beta oscillations. On the other hand, the reduction of alpha and beta activity in the region that was relevant to the task lead to the disinhibition of cortical activity, shaping the functional network. In support of this, 1 previous study showed that if beta oscillations in the motor cortex were maintained at high levels, by using alternating transcranial current stimulation, slow voluntary movement was observed in a visuo-motor tracking task [45]. Desynchronization of the beta oscillation band is needed for normal motor movement, the failure of which is correlated with motor deficits [46]. Previous studies have shown that during spatial attention [47,48] and working memory [49,50] tasks, oscillations in the alpha frequency band decrease in regions related to a given task, but increase in regions that are task-irrelevant. The network underlying consciousness in our study is compatible with the theory supported by these previous reports. One possible interpretation is that large-scale interactions in the second and third components might be associated with allocation of neural resources in the brain network to aid in shaping the functional architecture of the conscious state [43]. 

## Conclusions

This study identified 4 network components for the conscious and unconscious states, each with unique patterns for large-scale interactions in the spectral domain. Our results indicate that the difference between the conscious and unconscious states is characterized more by the switching of specific frequency modes in the large-scale communication occurring in entire cortical regions, rather than the disconnection of interactions amongst particular cortical regions.

## Materials and Methods

### Subjects and Materials

All experimental and surgical procedures were performed in accordance with the experimental protocols (No. H24-2-203(4)) approved by the RIKEN ethics committee and the recommendations of the Weatherall report, "The use of non-human primates in research". Implantation surgery was performed under sodium pentobarbital anesthesia, and all efforts were made to minimize suffering. No animal was sacrificed in this study. Overall care was managed by the Division of Research Resource Center at RIKEN Brain Science Institute. The animal was housed in a large individual enclosure with other animals visible in the room, and maintained on a 12:12-h light:dark cycle. The animal was given food (PS-A; Oriental Yeast Co., Ltd., Tokyo, Japan) and water ad libitum, and also daily fruit/dry treats as a means of enrichment and novelty. The animal was occasionally provided toys in the cage. The in-house veterinary doctor checked the animal and updated daily feedings in order to maintain weight. We have attempted to offer as humane treatment of our subject as possible.

Neural and behavioral recordings were performed by employing a multi-dimensional recording technique [13]. Chronically implanted, customized multichannel ECoG electrode arrays (Unique Medical, Japan) were used for neural recording [13]. Electrodes were made of 3-mm diameter platinum discs that were dimpled at the center after being exposed to an insulating silicone sheet 0.8 mm in diameter. The array was implanted in the subdural space in 4 adult macaque monkeys（M1-M3 are *Macaca fuscata* and M4 is *Macaca mulatta*). One hundred and twenty-eight channel ECoG electrodes with an interelectrode distance of 5 mm were implanted in the left hemisphere, continuously covering over the frontal, parietal, temporal, and occipital lobes (Figure 1A and S1). Additionally the electrodes of Monkey M1 covered the medial frontal and parietal walls and the electrodes of Monkey M2 covered the medial frontal and occipital walls. Reference electrodes were made of rectangular platinum plates placed in the subdural space between the ECoG array and dura mater. Lastly, ground electrodes were placed in the epidural space (See 13 for the detailed method). Parts of the dataset are shared in the public server Neurotycho.org (http://neurotycho.org/) [13].

## Experimental Procedures

### Anesthesia experiment

We did 2 experiments in which the monkey was under anesthesia. Two anesthetic agents, ketamine and medetomidine, were used in the first experiment. In the second experiment, only propofol was used (See Table S1). 

During the experiments, the monkey was seated in a primate chair with both arms and head movement restricted. The monkey’s eyes were covered to refrain from evoking visual response during the entirety of the experimental period. In the experiment using propofol-induced anesthesia, electromyography (EMG) was performed at a sampling rate of 1 kHz by a Cerebus data acquisition system. This was accomplished by putting 2 electrodes (Nihon Kohden, Disposable electrode for ECG Monitoring M-150) over the top of each hand and monitoring finger movement. The difference in potential between the 2 electrodes was used as the EMG signal. Neural data were also acquired during the awake and anesthetized conditions. In the awake condition, a monkey would sit calmly for up to 20 min at which time we would start to monitor heart rate. In experiments where the ketamine-medetomidine cocktail was used to induce anesthesia, the ketamine (^~^5.0 mg/kg for M1-M3, 8.8 mg/kg for M4) and medetomidine (^~^0.016 mg/kg for M1-M3, 0.053 mg/kg for M4) were injected intramuscularly (see Table S1). In contrast, propofol-induced anesthesia was achieved through intravenous propofol (5.2 mg/kg for M1, M2) injection (see Table S1). LOC was defined as the point at which a monkey no longer responded to manipulation of the monkey’s hand or touching of the nostril or philtrum with a cotton swab. As an additional confirmation that the monkey had achieved LOC, we could observe slow wave oscillations in the neural signal. After LOC was established, neural activity was recorded for ~25 min for the ketamine and medetomidine-induced anesthesia experiment and ~10 min for the propofol-induced anesthesia experiment. Heart rate and breathing were monitored carefully throughout the length of the experiment. We performed two to three recording sessions for each monkey, all on separate days (See Table S1).

### Natural sleep experiment

During the sleep experiment under the conditions of sleep, awake with eyes closed, and awake with eyes open, the state of the monkey was monitored by recording electrooculogram (EOG) and EMG signals. EOG signals were recorded from the muscles of the right eye by placing 2 electrodes on near the outer and inner canthi of the right eye. The difference in potential between the 2 electrodes was then used as the EOG signal. EMG signals were recorded from the chin and top of the right and left hands. Two electrodes were put on the chin, and the difference in the potential between these electrodes was used as the EMG signal. Lastly, the EMG signal for over the hands was collected as previously described.

In the sleep condition, the eyes were covered by an eye mask, and the experimental room was kept quiet and dark for up to 1.5 hours. During this period, slow wave oscillation appeared intermittently in the ECoG signal. Immediately following the sleep condition, the light was turned on, and data acquisition was continued under the eyes closed condition for 10 minutes. The eye mask was removed for the eyes open condition, which lasted for 10 minutes. For monkeys M1 and M2, 3 and 4 recording sessions were performed on separate days, respectively (See Table S1). The sleep state was defined by the degree of spatial synchronization in slow wave oscillations (See Text S1, Figure S2 and S3 for the detail method).

### Signal processing and spectral Granger causality analysis

ECoG signals were recorded at a sampling rate of 1 kHz by a Cerebus data acquisition system (Blackrock, UT, USA) and down-sampled to 200 Hz. The differential activity between nearest neighbor electrode pairs was calculated as re-referenced local cortical activity (See Figure 1A and S1). Each original electrode signal was used only once for re-referencing so that each re-referenced signal was created from unique electrode pairs. In other words, the total number of electrodes that were re-referenced was half of the original electrode number. Throughout the manuscript, the re-referenced electrode and signal will be referred to as “electrode” and “signal.”

We adapted a Matlab software package, the “Granger Causal Connectivity Analysis” (GC toolbox) developed by Seth [15], to calculate spectral Granger causality. For all experiments, ECoG signals were converted to 2-sec time series bins without overlap, and 60 (120 sec) bins were randomly selected from all recording sessions for each condition. In the 2 anesthesia experiments (ketamine-medetomidine and propofol), 60 (120 second) bins were selected from all recording sessions for each condition. For instance, in the case of M3 with 3 days of recording, 20 bins (total 40 seconds for each of the awake and the anesthetized conditions) were randomly selected from the data of each recording session. By pooling data for 3 days, the total number of bins used for the monkey was 60 bins (120 sec) for each condition. In the natural sleep experiment, 60 (120 sec) bins in which the spatial synchronization index (SSI, see Text S1 for the detail) exceed the threshold (=0.25) were selected from all recording sessions for each condition. 

Bins with noisy signal were excluded (See Text S2). Multi-taper filtering was applied to each of the 2-sec bins to remove line noise at 50 Hz [51]. Next, the time series in the 2-sec bin was converted to finer time bins separated by 200 ms with no overlap (total 600 short bins for each of the awake and anesthetized/sleep conditions). In each of the 200-ms bins, the time series data was detrended by removing the best-fit linear trend, and transformed into z-score by subtracting its mean and then dividing by its standard deviation (Figure 1B). 

To test the accuracy of this result, we made 6 sample conditions using the same technique as described above. There was no overlap in the selected time bin amongst the different condition samples, and the bootstrap resampling method was applied. This procedure involved creating 100 bootstrap samples, in which the original data (600 short bins) was randomly resampled with short bins, while preserving serial order and causal relations amongst electrodes in the short bins.

For all bootstrap samples in all sample conditions, spectral Granger causality was calculated (Figure 1B). To detect the optimal model order for the bivariate mode of Granger causality, we plotted the minimum of the Akaike Information Criterion (AIC) as a function of the model order. We used the function “cca_find_model_order_mtrial” in the GC toolbox for all combinations of electrode pairs. In this study, the AIC monotonically decreased with increasing model order, up to a value of 15 for all combinations of electrode pairs (Figure S4A). However we found consistent results in the 4 model orders by comparing the data produced by using different models of order 5, 7, 10, and 15. Thus, we used a model order of 7 (35 ms) due to a balance between computational power and time. The covariance stationarity was checked by Kwiatkowski-Phillips-Schmidt-Shin (KPSS) test [15,52]. We used the function “cca_kpss_mtrial” in the GC toolbox and found that the time-series of all electrodes passed the KPSS test (Figure S4B). Each of the 200-ms bins was considered as an independent common stochastic process [53] and thus was used to construct a single model. We assumed that there were cortical regions with unique and sustained interactions for over 120 sec. In practice, the GC toolbox supports multi-trial functions, “cca_pwcausal,” which estimate a single model directly from the multi-trial data. In this analysis, each short bin was regarded as a different trial, and a single model was constructed from the pseudo multi-trial data.

Bivariate Granger causality was derived by using the spectral matrix for all combinations of electrode pairs [15,16,54] and for all frequency windows (size 2 Hz) ranging from 6 to 100 Hz. The lower limit of the frequency corresponded to 1 full cycle of oscillations with 200-ms short bins. For each electrode pair, 48 causal models were constructed.

### Support Vector Machine (SVM)

We used the Matlab software package “LIBLINEAR” for SVM [17]. In SVM, we used the default parameter setting in which the type of solver was “L2-regularized L2-loss support vector classification” and cost-value was set to 1. 

### Parallel factor analysis (PARAFAC)

We used the Matlab software package “N-way Toolbox “ [19] for PARAFAC. The type of constraint for each dimension was set to non-negativity. The proper number of components was determined by using the Core consistency diagnostic (CCD) in which number of components is highest when the value of CCD was kept to be more than 40% [20]. 

## Supporting Information

Figure S1 **The original and bipolar re-referenced ECoG electrode arrays on the left cortical surface of the 4 monkeys (M1-M4).** The white circles are the original electrodes, and red circles are bipolar re-referenced electrodes.
(TIF)

Figure S2 **The typical time series of normalized ECoG, EOG, EMG, and SSI in the sleep and eyes-closed conditions.** Three electrodes were selected for normalized ECoG1 to ECoG3. For preprocessing, the bandpass filter (fourth Butterworth filter) from 0.5 Hz to 100 Hz was applied to the ECoG signal, and the filtered signal was normalized by using its mean and standard deviation (Normalized ECoG). For EOG and EMG signals, the signals were converted to 1-sec time series bins with 200-msec overlap. The absolute value of signals was averaged for each bin, and the averaged value of the bin was normalized by using its mean and standard deviation (Normalized EOG and EMG).
(TIF)

Figure S3 **A: The density of the number of time bins for SSI, normalized EOG, and EMG for the sleep (red), eyes-closed (blue), and eyes-open (green) conditions, for monkeys M1 and M2.** B: The density of the number of time bins in relation with SSI and normalized EOG/EMG for the sleep, eyes-closed, and eyes-open conditions for monkeys M1 and M2.
(TIF)

Figure S4 **A: Normalized AIC for the awake and ketamine–medetomidine-induced anesthetized conditions for the 4 monkeys.** In 1 condition sample, the AIC was calculated for all combinations of electrode pairs. The AIC was averaged for all combinations of electrode pairs and was normalized by its mean and standard deviation along frequency. The blue and red lines indicate the averaged distribution of 4 monkeys for awake and anesthetized conditions, respectively. The range from minimum to maximum values is shown by the shading around the plots.
B: The pass rate of KPSS test for the awake and ketamine–medetomidine-induced anesthetized conditions for the 4 monkeys. In 1 condition sample, KPSS test was applied for all electrodes. The numbers of electrode that pass the KPSS test were averaged for all number of electrodes. The blue and red lines mean the averaged distribution of 4 monkeys for awake and anesthetized conditions, respectively. The range from minimum to maximum values is shown by the shaded areas around the plots.
(TIF)

Figure S5 **Individual consistency of the region score matrix amongst the 4 monkeys for all 4 components, and for the awake (red) and anesthetized (blue) conditions.** For each component (4 × 2 = 8), the spearman correlation coefficient of the region score matrix was calculated for all combinations of monkey pairs (6 pairs). Individual consistency was defined by the ratio of the number of significant correlations in monkey pairs to the number of all combinations of monkey pairs (p < 0.05, FDR correction).
(TIF)

Table S1 **Detailed information of the 3 experiments, namely, ketamine–medetomidine- and propofol-induced anesthesia and natural sleep experiments.**
(TIF)

Text S1 **Definition of sleep state.**
(DOCX)

Text S2 **Filtering of noisy ECoG signal.**
(DOCX)
