# Correction: Large-Scale Information Flow in Conscious and Unconscious States: an ECoG Study in Monkeys

**DOI:** 10.1371/journal.pone.0091438

**Published:** 2014-03-04

**Authors:** The PLOS ONE Staff

In the Results section, there is an equation missing from the second paragraph of the section entitled “Universality for different unconscious states (anesthesia and sleep)”. The correct equation and statement should read:

“To prove the similarity, we defined a spatial-frequency correlation as:
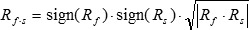
in which R_f_ is the spearman correlation coefficient of scores of components in the frequency dimension between the ketamine–medetomidine and 3-LOC experiments. R_s_ is the spearman correlation coefficient of scores of components in the electrode-pair dimension between the ketamine–medetomidine and 3-LOC experiments.”
