# Supplementary figures and images for: Large-Scale Information Flow in Conscious and Unconscious States: an ECoG Study in Monkeys
Source: PLoS One. 2013 Nov 15;8(11):e80845. doi: 10.1371/journal.pone.0080845 (PMC3829858; doi:10.1371/journal.pone.0080845)

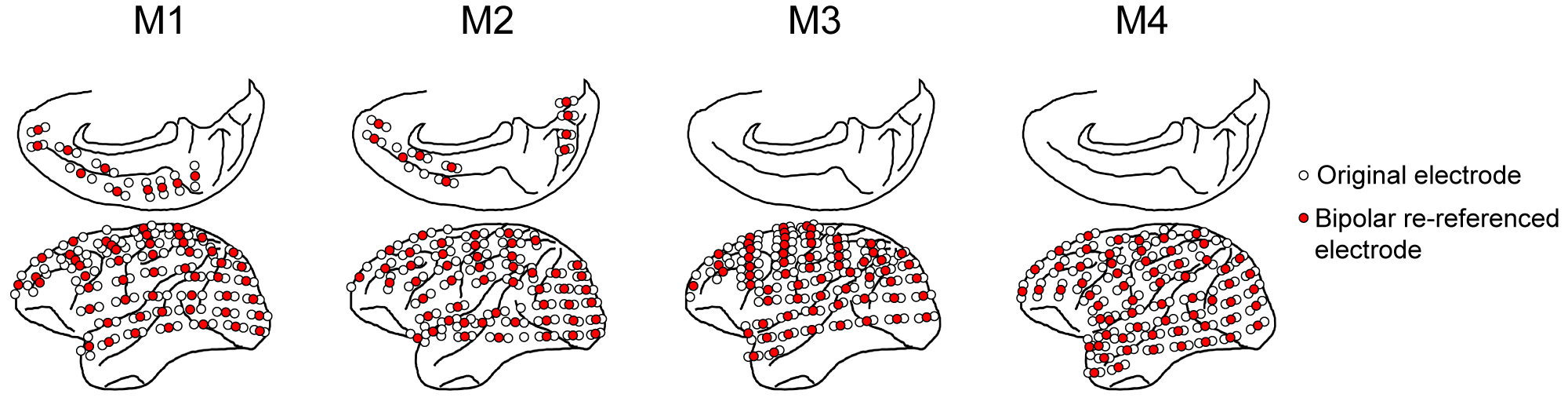

Supplement: Figure S1 — The original and bipolar re-referenced ECoG electrode arrays on the left cortical surface of the 4 monkeys (M1-M4). The white circles are the original electrodes, and red circles are bipolar re-referenced electrodes. (TIF) [file pone.0080845.s001.tif]

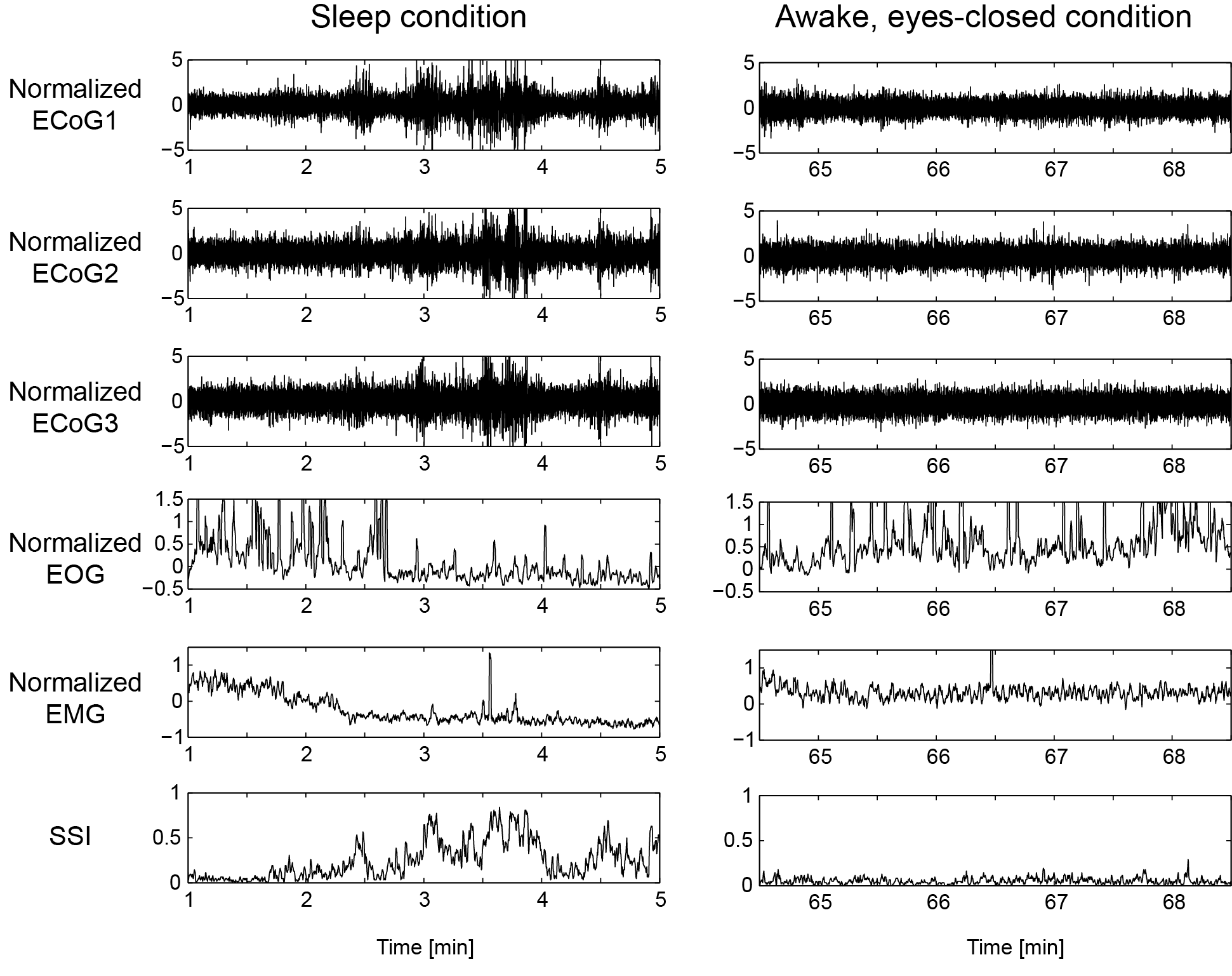

Supplement: Figure S2 — The typical time series of normalized ECoG, EOG, EMG, and SSI in the sleep and eyes-closed conditions. Three electrodes were selected for normalized ECoG1 to ECoG3. For preprocessing, the bandpass filter (fourth Butterworth filter) from 0.5 Hz to 100 Hz was applied to the ECoG signal, and the filtered signal was normalized by using its mean and standard deviation (Normalized ECoG). For EOG and EMG signals, the signals were converted to 1-sec time series bins with 200-msec overlap. The absolute value of signals was averaged for each bin, and the averaged value of the bin was normalized by using its mean and standard deviation (Normalized EOG and EMG). (TIF) [file pone.0080845.s002.tif]

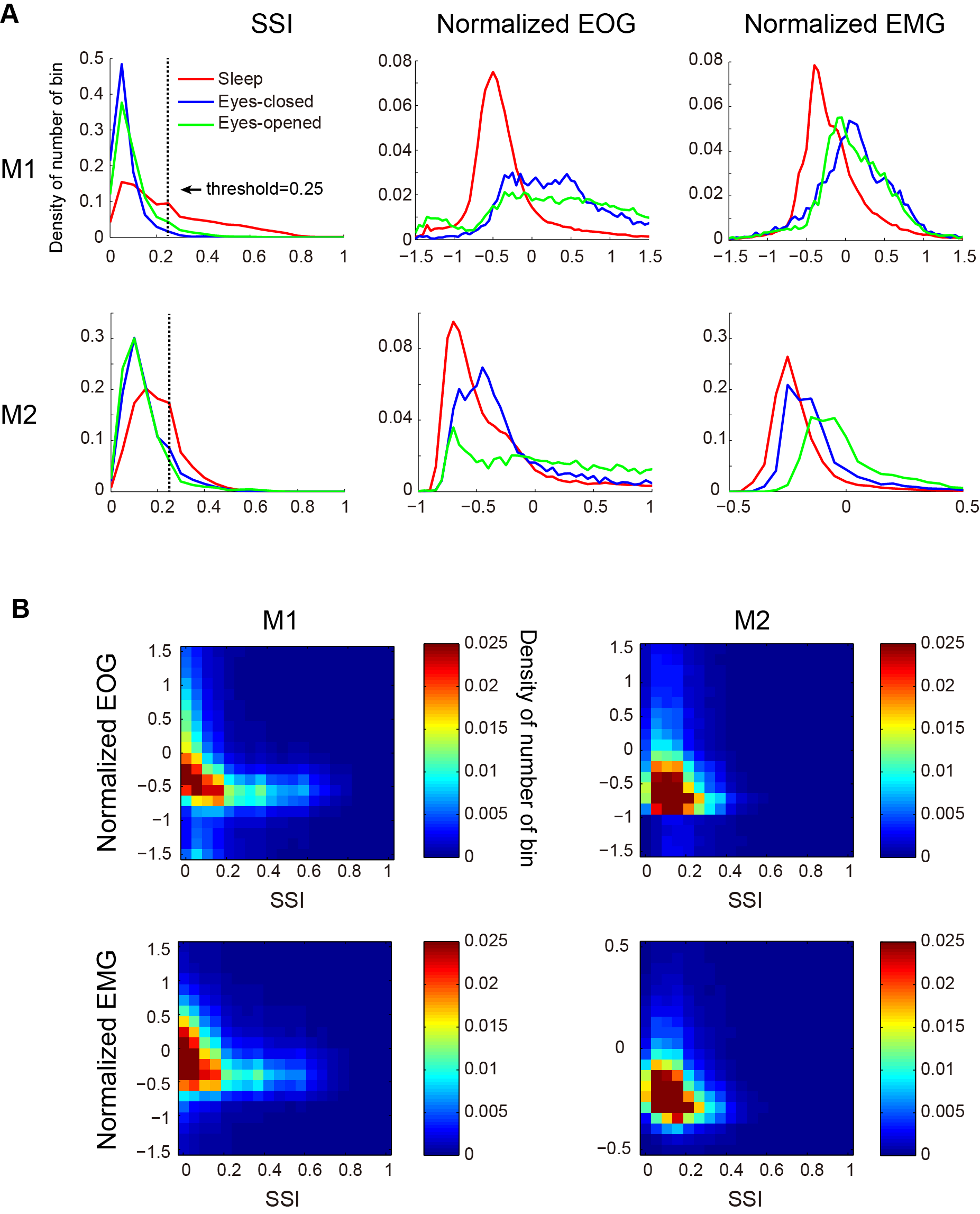

Supplement: Figure S3 — A: The density of the number of time bins for SSI, normalized EOG, and EMG for the sleep (red), eyes-closed (blue), and eyes-open (green) conditions, for monkeys M1 and M2. B: The density of the number of time bins in relation with SSI and normalized EOG/EMG for the sleep, eyes-closed, and eyes-open conditions for monkeys M1 and M2. (TIF) [file pone.0080845.s003.tif]

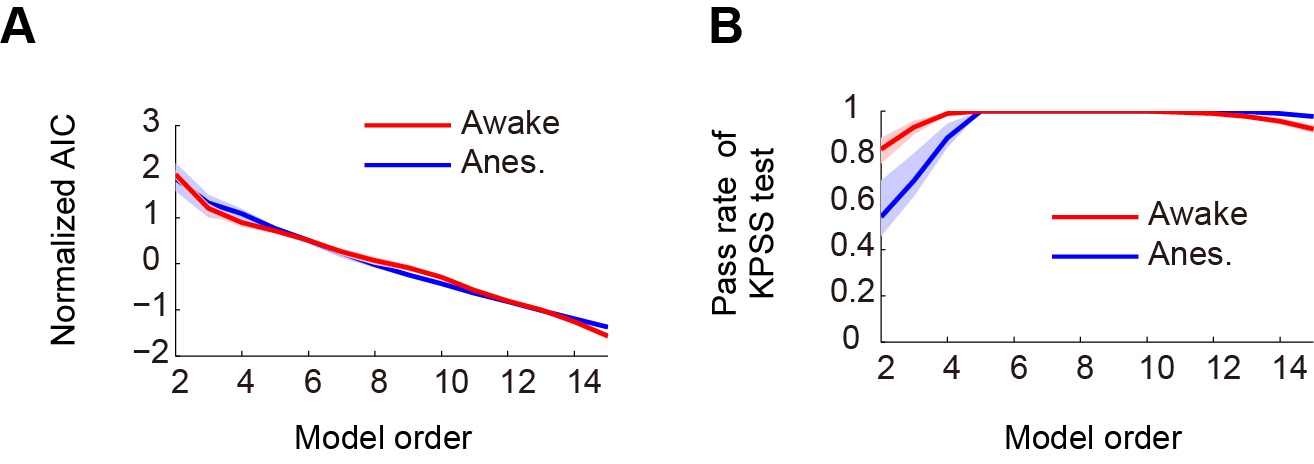

Supplement: Figure S4 — A: Normalized AIC for the awake and ketamine–medetomidine-induced anesthetized conditions for the 4 monkeys. In 1 condition sample, the AIC was calculated for all combinations of electrode pairs. The AIC was averaged for all combinations of electrode pairs and was normalized by its mean and standard deviation along frequency. The blue and red lines indicate the averaged distribution of 4 monkeys for awake and anesthetized conditions, respectively. The range from minimum to maximum values is shown by the shading around the plots. B: The pass rate of KPSS test for the awake and ketamine–medetomidine-induced anesthetized conditions for the 4 monkeys. In 1 condition sample, KPSS test was applied for all electrodes. The numbers of electrode that pass the KPSS test were averaged for all number of electrodes. The blue and red lines mean the averaged distribution of 4 monkeys for awake and anesthetized conditions, respectively. The range from minimum to maximum values is shown by the shaded areas around the plots. (TIF) [file pone.0080845.s004.tif]

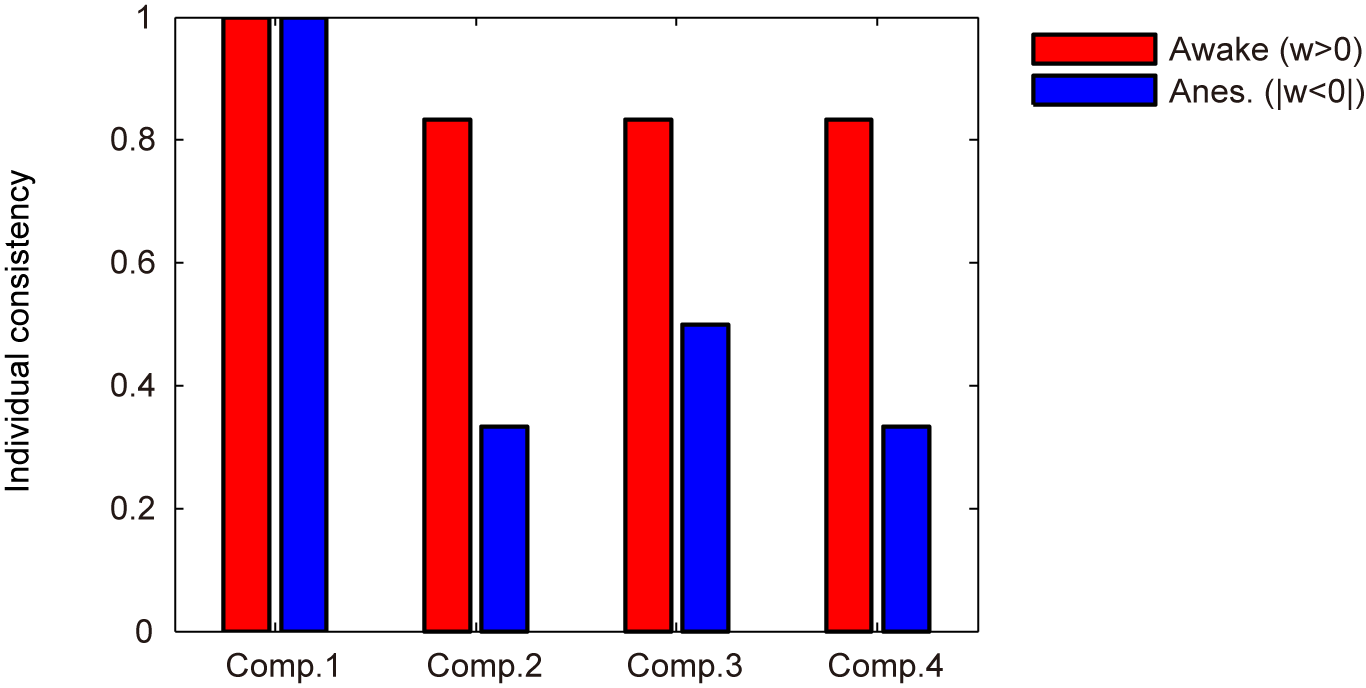

Supplement: Figure S5 — Individual consistency of the region score matrix amongst the 4 monkeys for all 4 components, and for the awake (red) and anesthetized (blue) conditions. For each component (4 × 2 = 8), the spearman correlation coefficient of the region score matrix was calculated for all combinations of monkey pairs (6 pairs). Individual consistency was defined by the ratio of the number of significant correlations in monkey pairs to the number of all combinations of monkey pairs (p < 0.05, FDR correction). (TIF) [file pone.0080845.s005.tif]

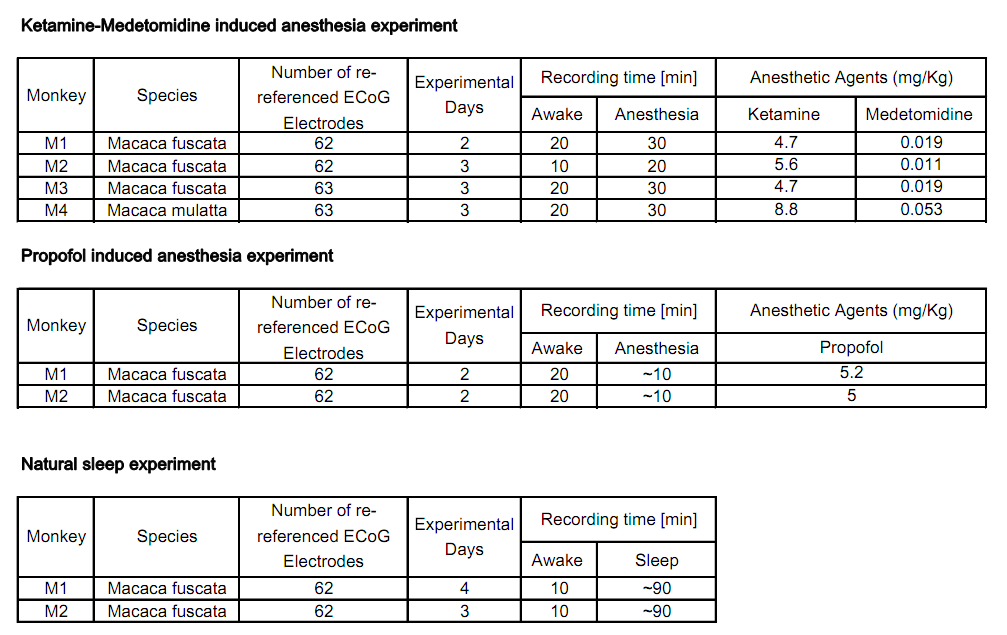

Supplement: Table S1 — Detailed information of the 3 experiments, namely, ketamine–medetomidine- and propofol-induced anesthesia and natural sleep experiments. (TIF) [file pone.0080845.s006.tif]
