# Supplementary material for: Large-Scale Information Flow in Conscious and Unconscious States: an ECoG Study in Monkeys
Source: PLoS One. 2013 Nov 15;8(11):e80845. doi: 10.1371/journal.pone.0080845 (PMC3829858; doi:10.1371/journal.pone.0080845)
Supplement: Text S1 — Definition of sleep state. (DOCX) [file pone.0080845.s007.docx]

**Text S1. Definition of sleep state**

During the sleep condition, we were unable to locate the sleep spindle and k-complex in the ECoG signal, which is needed to define the stage of sleep. However, we found a correlation between the appearance of slow wave oscillations in the ECoG signal and a decrease of EOG and EMG signals. Therefore, we developed a method to estimate the sleep state by quantifying the spatial synchronization of slow wave oscillation in the cortex.

The bipolar re-referenced ECoG signal was converted to 1-sec time series bins with 200-msec time shifts for spectral analysis. We used a Matlab software package, chronux, for multi-taper spectrum estimation [[1](#_ENREF_1)]. The ECoG spectrum power was estimated on each 1-sec bin with 2-Hz resolution using 5 Slepian data tapers. Estimated frequency bands ranged from 1 to 4 Hz. For all electrodes, the spectral power from 1 to 4 Hz was averaged for each time bin (delta band power).

To detect slow wave oscillations, the delta band power in the sleep condition was compared with that in eyes-open condition. For this, the delta band power of each time bin in the 3 conditions was normalized by the median value of the delta band power of the time bins in the eyes-open condition. The normalized delta band power of each time bin was binarized by an arbitrarily determined threshold, which was set to be 3. To evaluate how much the slow wave oscillation occurred on the surface of the cortex, we defined the spatial synchronization index (SSI) of delta band power by averaging the number of the binarized delta band power for all electrodes. If SSI at 1 time bin was 0.5, then half of the electrodes had a delta band power 3 times larger than the median value of delta band power in the eyes-open condition.

Figure S2 shows the typical time series of ECoG, EOG, EMG, and SSI. In the sleep condition, increased SSI indicated that the spatially synchronized slow wave oscillation occurred over the cortex, and the normalized EOG and EMG decreased. On the other hand, in eyes-closed condition, normalized ECoG and EMG signals were maintained higher and the SSI was maintained lower. The density of the time bin of the SSI, normalized EOG, and EMG was calculated for the sleep, eyes-closed, and eyes-open conditions (Figure S3A). Data was then averaged for all experimental days. Monkey M2 had 3 experimental days; however, the recording of EOG and EMG signals failed in 1 experiment. Thus, the data of the other 2 experiments in monkey M2 were used in Figure S3. Figure S3A shows a trend of a higher SSI and lower EOG and EMG in the sleep condition than in the eyes-open and eyes-closed conditions.

In Figure S3B, the density of the number of bins in relation with the SSI and normalized EOG/EMG was plotted for the sleep, eyes-closed, and eyes-open conditions. We defined the monkey as being in the sleep state when the SSI was more than 0.25, in which the normalized EOG and EMG signals were maintained at the lower level (Figure S3B). The density of the number of bins in the sleep state during the sleep condition was 48% in M1 and 19% for M2. We estimate that our sleep state corresponded approximately to the sleep stages 3 and 4 in which slow wave oscillation occurs and there is no eye movement or muscle activity.

1. Mitra P, Bokil H (2008) Observed Brain Dynamics. New York: Oxford University Press.
