# Supplementary material for: Large-Scale Information Flow in Conscious and Unconscious States: an ECoG Study in Monkeys
Source: PLoS One. 2013 Nov 15;8(11):e80845. doi: 10.1371/journal.pone.0080845 (PMC3829858; doi:10.1371/journal.pone.0080845)
Supplement: Text S2 — Filtering of noisy ECoG signal. (DOCX) [file pone.0080845.s008.docx]

**Text S2. Filtering of noisy ECoG signal**

Signals from all electrodes were visually inspected, and the electrodes that had predominately poor signal-to-noise properties were excluded from further analysis. On average, 1 electrode per recording session was excluded. In addition, the artifact in the shorter segments was excluded. In the ketamine–medetomidine-induced anesthesia experiment, the time series of the grand average was calculated for each condition. The grand averaged time series was then converted to 2-sec time series bins without overlap. If a bin contained values that exceeded the standard deviation of the grand averaged series by 4×, that bin along with the previous and next bins were excluded (2% in the awake condition, 0.5% in the anesthetic condition) from further analysis. In the propofol-induced anesthesia and natural sleep experiments, if the EMG signal recorded from the top of the right and left hands exceeded the baseline level during the time when the monkey did not move, the bin containing the incident along with the previous and next bins were excluded for further analysis (the propofol-induced anesthesia experiment: 14% in the awake condition, 2% in the anesthetic condition, the natural sleep experiment: 11% in the awake condition, 17% in the sleep condition).
